# Intracellular HIV-1 Tat regulator induces epigenetic changes in the DNA methylation landscape

**DOI:** 10.3389/fimmu.2025.1532692

**Published:** 2025-03-04

**Authors:** Andrea Rodríguez-Agustín, Rubén Ayala-Suárez, Francisco Díez-Fuertes, María José Maleno, Izar de Villasante, Angelika Merkel, Mayte Coiras, Víctor Casanova, José Alcamí, Núria Climent

**Affiliations:** ^1^ AIDS and HIV Infection Group, Fundació de Recerca Clínic Barcelona-Institut d’Investigacions Biomédiques August Pi i Sunyer (FRCB-IDIBAPS), Barcelona, Spain; ^2^ Universitat de Barcelona (UB), Barcelona, Spain; ^3^ AIDS Immunopathology Unit, Centro Nacional de Microbiología, Instituto de Salud Carlos III (ISCIII), Madrid, Spain; ^4^ Centro de Investigación Biomédica en Red sobre Enfermedades Infecciosas (CIBERINFEC), Instituto de Salud Carlos III (ISCIII), Madrid, Spain; ^5^ Bioinformatics Unit, Josep Carreras Leukaemia Research Institute (IJC), Badalona, Spain; ^6^ Immunopathology and Viral Reservoir Unit, Centro Nacional de Microbiología, Instituto de Salud Carlos III (ISCIII), Madrid, Spain

**Keywords:** HIV infection, Tat, epigenetics, DNA methylation, inflammation

## Abstract

**Introduction:**

The HIV regulatory protein Tat enhances viral transcription and also modifies host gene expression, affecting cell functions like cell cycle and apoptosis. Residual expression of Tat protein is detected in blood and other tissues even under antiretroviral treatment. Cohort studies have indicated that, despite virologic suppression, people with HIV (PWH) are at increased risk of comorbidities linked to chronic inflammation, accelerated immune ageing, and cellular senescence, sometimes associated with abnormal genomic methylation patterns. We analysed whether Tat influences DNA methylation and subsequently impacts the transcriptional signature, contributing to inflammation and accelerated ageing.

**Methods:**

We transfected Jurkat cells with full-length Tat (Tat101), Tat’s first exon (Tat72), or an empty vector (TetOFF). We assessed DNA methylation modifications via the Infinium MethylationEPIC array, and we evaluated transcriptomic alterations through RNA-Seq. Methylation levels in gene promoters or body regions were correlated to their expression data, and subsequently, we performed an overrepresentation analysis to identify the biological terms containing differentially methylated and expressed genes.

**Results:**

Tat101 expression caused significant hyper- and hypomethylation changes at individual CpG sites, resulting in slightly global DNA hypermethylation. Methylation changes at gene promoters and bodies resulted in altered gene expression, specifically regulating gene transcription in 5.1% of differentially expressed genes (DEGs) in Tat101- expressing cells. In contrast, Tat72 had a minimal impact on this epigenetic process. The observed differentially methylated and expressed genes were involved in inflammatory responses, lipid antigen presentation, and apoptosis.

**Discussion:**

Tat expression in HIV infection may constitute a key epigenetic modelling actor that contributes to HIV pathogenesis and chronic inflammation. Clinical interventions targeting Tat blockade may reduce chronic inflammation and cellular senescence related to HIV infection comorbidities.

## 1 Introduction

The human immunodeficiency virus type 1 (HIV-1) transcriptional transactivator (Tat) is an early expressed viral protein that boosts the viral replication cycle (1). Tat enhances the elongation of HIV-1 nascent transcripts recruiting the cellular positive transcription factor (P-TEFb) to the transactivation response element (TAR), located at the non-coding regions of the 5′ and 3′ ends of the proviral genome (2). P-TEFb mediates the hyperphosphorylation of the RNA polymerase II, increasing its activity and ensuring the transcription of full-length viral mRNAs. Tat protein is encoded by two exons: the first encodes residues 1 through 72, producing a functional domain of Tat form known as Tat72, which enables HIV-1 replication through TAR-dependent activation of transcription. The second exon encodes residues 73 through 101 and has been noted as genetically diverse and non-essential for Tat’s primary function in HIV-1 replication (2). However, full-length Tat expression is conserved across all HIV strains, and the Tat101 form is much more prevalent in people with HIV (PWH) than their truncated variants Tat72 and Tat86, indicating its biological significance (3, 4). Indeed, Tat101 present a greater number of functionalities, allowing a more efficient viral replication in macrophages (5), altering actin polymerisation (4) or mitochondrial function (6), or promoting viral persistence protecting infected cells from apoptosis (7). Therefore, in addition to its canonical function as a transactivator of HIV transcription, Tat exerts alternative roles crucial for viral cycle progression and immunopathogenesis. These functions include host gene expression regulation through the interaction with transcription factors, i.e., nuclear factor κB (NF-κB), NFAT, or Sp1, that control gene expression including proinflammatory cytokines and proteins regulating the immune response (7, 8). Tat regulates gene expression directly and indirectly, eliciting the expression of inflammatory cytokines and chemokines in different cell types. Tat101 induces the hyperacetylation of NF-κB, which induces T-cell activation genes and cytokine regulation (9). Tat101 also promotes the expression of IL-2 in Jurkat T cells, stimulating T-cell survival, proliferation, and immune responses (9). Evidence shows that Tat directly interacts with promotor regions of some genes (10). Additionally, host gene regulation may occur via Tat interactions with transcription factors (10, 11) or by direct binding to cellular mRNAs (12).

Epigenetics comprises several molecules and mechanisms that alter gene expression during a long-term period in the context of the same DNA sequence (13). These mechanisms include DNA methylation and the covalent addition of a methyl group to the fifth carbon of cytosine (14). In mammals, this modification is most observed in the context of CpG dinucleotides, which themselves are found in high frequency in DNA regions known as CpG islands (CGIs) (15). Notably, 72% of gene promoters found in the human genome contain CGIs and are located within transcription start sites (TSSs) (15, 16). High levels of 5-methylcytosine in gene promoters are linked to transcriptional suppression since the hypermethylation leads to the recruitment of gene expression suppressor proteins, reduction of the interaction between DNA and transcription factors, and chromatin compaction (15). In contrast, gene body methylation positively correlates with gene expression, helping to avoid spurious transcription initiation, regulating alternative splicing, and altering transcription elongation (17–19). Accordingly, DNA methylation in different genome regions and gene features is strongly associated with gene transcription regulation.

HIV infection induces significant changes in the host epigenome, leading to a cellular environment more favourable to its replication and persistence (20). Various authors have reported that HIV-1 infection leads to an increment in the average genome methylation status, although both hypermethylation and hypomethylation changes are observed in the host genome (21–26). Alterations in DNA methylation have been associated with cellular senescence, chronic inflammation, and accelerated biological ageing in PWH, conditions associated with decreased life expectancy (27–31). Even though antiretroviral therapy (ART) can partially reverse HIV-induced DNA methylation changes, an increase in comorbidities associated with HIV infection is still detected (21). It has been described that Tat promotes the overexpression of DNA (cytosine-5)-methyltransferase 1 (DNMT1), DNMT3A, and DNTM3B, which are responsible for adding methyl groups, suggesting a role for Tat in DNA methylation remodelling (32–34). Tat expression is detected in the serum and central nervous system from PWH even under ART treatment (35, 36). Our own unpublished preliminary work and others have described that intracellular Tat expression can induce cellular changes related to cellular senescence such as neuroinflammation, oxidative stress, mitochondrial dysfunction, cell cycle arrest, and apoptosis resistance (6, 7, 33, 37, 38). However, there is no prior research interrogating whether Tat induces methylation changes at specific sites across the genome and the possible impact on the development of age-related comorbidities.

We hypothesised that the expression of Tat induces epigenetic changes at the DNA methylation level, driving the expression of genes potentially involved in cellular senescence. We have shown that full-length Tat induces hypermethylation and hypomethylation changes in individual CpGs. The main proportion of these methylation changes is found in clusters associated with promoter and gene body sequences, leading to expression changes in genes that participate in inflammatory response or apoptosis-related pathways. Gaining knowledge on how intracellular full-length Tat alters the DNA methylation landscape would provide a better understanding of HIV pathogenesis and the induction of a senescence program in Tat-expressing cells.

## 2 Material and methods

### 2.1 Cells

Jurkat HIV-Tat stable transfectants were generated in and obtained from Alcamí and Coiras’ lab (Instituto de Salud Carlos III, Madrid, Spain) (7). Briefly, the Jurkat TetOFF cell line from Clontech (Mountain View, CA, USA) was stably transfected with a pTRE2hyg plasmid encoding full-length HIV-Tat cDNA (101aa; Tat101), a pTRE2hyg plasmid encoding only the first exon of HIV-Tat (72aa; Tat72), or an empty pTRE2hyg vector as a control (TetOFF). The expression and function of HIV-Tat in this cellular model have been extensively characterised by previous studies (4, 6, 7, 39). In this model, the administration of 1 μg/mL doxycycline (Takara Bio, Mountain View, CA, USA; Ref. 631311) to the culture medium for 48 h effectively resulted in the repression of Tat expression as previously described (4). Cells were cultured in RPMI 1640 medium with l-glutamine supplemented with 10% (v/v) foetal bovine serum (FBS) (Biowest, Nuaillé, France; Ref. S181H), 100 μg/mL streptomycin, and 100 U/mL penicillin (BioWhittaker, Walkersville, MD, USA) at 37°C and 5% CO_2_. To stabilise these cell lines, 300 μg/mL hygromycin B (BD Biosciences, Clontech) and 300 μg/mL geneticin (Sigma-Aldrich, St. Louis, MO, USA) were added to culture media.

### 2.2 Methylome analysis

#### 2.2.1 Genome-wide DNA methylation profiling

A total of 4 × 10^5^ Jurkat cells per well with the different HIV-Tat constructs or the empty vector control (three biological replicates) were grown in the presence or absence of 1 μg/mL doxycycline for 2 days. Cell pellets were then obtained and frozen. Genomic DNA from the different cell lines was isolated using a QIAamp DNA Mini Kit (Qiagen, Hilden, Germany; Ref. 51304) following the manufacturer’s instructions; 600 ng of DNA quantified by Quant-iT™ PicoGreen™ (Invitrogen, Waltham, MA, USA; Ref. P7589) was used for bisulfite conversion with the EZ DNA Methylation kit (Zymo Research, Irvine, CA, USA; Ref. D5001). An incubation of 16 cycles of a two-step conditions: 95°C for 30 seconds, then 50°C for 1h, was performed for the Illumina Infinium Methylation Assay, according to the manufacturer’s instructions. Once converted, DNA was eluted in 15 μL of the provided Elution Buffer. Treated DNA samples were transferred to the Genomic Unit of the Josep Carreras Research Institute, Badalona, Spain, and were assayed using the Infinium MethylationEPIC BeadChip microarray v1.0 (Illumina, San Diego, CA, USA). All samples were analysed on the same microarray chip following the manufacturer’s instructions. Raw image intensities were obtained by scanning the microarrays using the HISCanSQ Analysis System (Illumina, San Diego, CA, USA).

#### 2.2.2 Differential methylation analysis

Methylation data analysis was performed using the EPIPE methylation analysis pipeline (https://github.com/ijcBIT/epipe). Briefly, raw DNA methylation data were processed using the *minfi* R package that applies background correction, dye-bias normalisation (Noob method selected), and estimates signal detection. Probes with low signal (*p-*value > 0.01), located in non-CpG methylation positions, positions annotated as single-nucleotide polymorphism, or CpG located at sex chromosomes were removed. After the quality control process and filtering, 812,253 DNA methylation positions remained for downstream analysis. DNA methylation levels (β-values) from each probe were computed as the ratio of methylated signal divided by the sum of methylated and unmethylated signal ranging from 0 (completely unmethylated) to 1 (completely methylated). The presence of differentially methylated CpG positions (DMPs) was evaluated through pairwise comparisons among Tat (Tat101 and Tat72) and empty vector (TetOFF) cell lines, including doxycycline treatment. Linear models were used for differential methylation analysis using the *Limma* R package, and the Benjamini–Hochberg-corrected false discovery rate (FDR) was used to adjust *p-*values after multiple tests. DMPs were defined as those with a cut-off of absolute difference in β-values greater than 50% (|Δβ| > 0.5) and an FDR-adjusted *p-*value < 0.05. To identify more biologically relevant changes at the methylome level, regions containing a group of proximal CpG with a differential mean of methylation between groups were studied, which were defined as differentially methylated regions (DMRs). The *DMRCate* R package was used for DMR identification, considering a DMR as a region of three or more CpG with a mean difference of β-value greater than 25% (|Δβ| > 0.25) and an FDR-adjusted *p-*value < 0.1 between experimental conditions. A sliding window of 1,000 nucleotides was applied in the search for DMRs.

#### 2.2.3 DMP and DMR functional analysis

To link DMPs and DMRs to their genomic functions, both were annotated using the *annotatr* and *org.Hs.eg.db* R packages with the Genome Reference Consortium Human Build 37 (GRCh37/hg19) Organism as reference. Annotations included two types of structures (**Supplementary Figure 1**): 1) gene features, which were grouped in three classes regarding gene function as TSS proximal zone [built from sequences defined as 1 to 5 kb upstream of the TSS, promoter, and 5′-untranslated region (UTR)], gene body (composed by sequences annotated as exons, introns, and 3′-UTR), and intergenic regions, and 2) CGI annotation, which includes CpG islands (sequences 0.2–1 kb long with a GC% > 0.5 and an observed versus expected CpG ratio > 0.6), CpG shores (<2 kb away from islands), CpG shelves (between 2 kb and 4 kb away from islands), and open sea (>4 kb away from islands). Overrepresentation analysis of biological terms enriched with DMR-containing genes was performed using the gsaregion function from the *missMethyl* R package (40) and using Gene Ontology, Kyoto Encyclopedia of Genes and Genomes (KEGG), Reactome, Transcription Factor Targets (TFT), Hallmarks from Molecular Signature DB, and WikiPathways databases as a reference. Biological terms are considered statistically significant with an FDR-corrected *p-*value < 0.1 and a ratio greater than 2% of genes with DMRs present in a term versus the total number of genes belonging to that term.

### 2.3 Transcriptomic analysis

#### 2.3.1 Library preparation and sequencing

RNA-Seq was performed to assess the variations at the transcriptional level elicited by the expression of the different HIV-Tat constructs. Ten million cells from the different HIV-Tat stable transfectants or TetOFF controls were harvested from three independent fresh cultures. Total RNA was isolated using the miRNeasy kit (Qiagen, Hilden, Germany; Ref. 217084) following the manufacturer’s instructions. Nucleic acid concentration was measured at 260 nm using a NanoDrop One (Thermo Fisher Scientific, Waltham, MA, USA) spectrophotometer. RNA integrity was evaluated using an RNA Chip Nano 6000 (Agilent, Madrid, Spain; Ref. 5067-1511) in a Bioanalyzer 2100 equipment (Agilent, Madrid, Spain), obtaining RNA integrity number (RIN) values between 9.6 and 10. Libraries for RNA-Seq were synthesised using the TruSeq Stranded Total RNA library prep kit with the Ribo-Zero Human (Illumina, San Diego, CA, USA; Ref. RS-122-2201) from 1 µg total RNA following the protocol provided by the manufacturer. Before cDNA synthesis, ribosomal RNA was depleted, and steps of cDNA purification and cleansing were performed using AMPure XP magnet beads (Beckman Coulter, Brea, CA, USA; Ref. A63882). The libraries were pooled (n = 12) and sequenced with a single NextSeq 500/550 High output kit v2.5 for 75 cycles (Illumina, San Diego, CA, USA; Ref. 20024906) in a NextSeq 500 sequencer (Illumina) in the Genomics Unit of the National Center for Microbiology in the Instituto de Salud Carlos III (Madrid, Spain). The expected output per sample was 33.3 million reads.

#### 2.3.2 Quality assessment of reads and transcriptome alignment

The quality of raw reads was assessed in the fastq files using the FastQC v0.11.9 software (http://www.bioinformatics.babraham.ac.uk/projects/fastqc/) to evaluate base quality, adapter presence, or overrepresented sequences. Trimming of poor-quality bases and adapters was carried out with the Trimmomatic tool (http://www.usadellab.org/cms/?page=trimmomatic) using a 4-base-wide sliding window to trim the ends when the average quality per base drops below 15 or any base with a Phred quality below 3. The software also cuts adapters from the sequences, allowing two seed mismatches. Any sequences shorter than 30 bases were removed from the output. The alignment was performed by Bowtie2 within the RSEM pipeline (https://github.com/deweylab/RSEM) following the developer’s guidelines. The reference index file of human transcriptome (GRCH38) and transcript-specific files were prepared with Bowtie2 employing the versions of the .gtf and .fasta files of the GRCh38 primary assembly from the Ensembl release 110. The reads that passed the quality control requirements defined above were aligned to the reference to estimate gene expression abundance in transcripts per million, calculating 95% credibility intervals.

#### 2.3.3 Differential expression and enrichment analyses

The estimated gene level expression was obtained using RSEM’s approximate maximum likelihood estimates through the annotated alignment of the reads in genomic coordinates. The gene-level expression was given in transcripts per million (TPM) as a relative measure that represents the number of copies each gene should have supposing the whole transcriptome contains exactly 1 million transcripts. The expected read count for each gene was used for differential expression analysis in pairwise comparisons between Jurkat cell lines. The R package *EBSeq* performs differential expression analysis with RNA-Seq data using an empirical Bayesian approach based on the negative binomial distribution. Genes were considered differentially expressed (DEG) when they had a |fold-change| > 2 between groups and a posterior probability of being differentially expressed (PPDE) greater than 0.95. An overrepresentation analysis was conducted using the DEG obtained in each comparison in the web-based application Kobas-i (41). The KEGG, Reactome, and Gene Ontology databases were included as references. A biological term was considered enriched with DEG when it contained at least two DEGs representing more than 2% of the total number of the genes comprising the term and an FDR-corrected *p-*value < 0.1.

### 2.4 Methylome and transcriptome integration analysis

To describe which gene’s expression is affected by the alteration of the methylation related to their gene bodies or regulating zones, we integrated the results from the methylome and transcriptomic datasets. We selected the DMRs from each comparison and defined that they belonged to the promoter of a gene if they were annotated as promoter or 5′UTR, or else to the gene body if they were annotated as coding sequence, first exons, exons, introns, or 3′UTR. We used Ensemble gene identifiers to link DMR and DEG information. Subsequently, we applied a Spearman’s correlation analysis with the cor.test function from the R package *stats* between the mean β-values of the DMRs for each sample and the TPM of their matching DEG. We considered that a gene is differentially methylated and expressed (DMEG) if it obtained a Spearman’s coefficient (ρ) >0.7 when correlations were produced between β-values in gene bodies and their expression level or ρ < −0.7 if the correlations were obtained between the β-values from promoters and their TPM. To elucidate which cellular processes are affected by the alteration of gene expression influenced by methylation changes, we performed an overrepresentation analysis using Kobas-i with the DMEG obtained in each comparison. Biological terms used for the overrepresentation analysis belong to the KEGG, Reactome, and Gene Ontology databases. Enriched terms are those with at least two DMEGs present in a ratio of DMEG between the number of genes that belong to that term greater than 2% and an FDR-corrected *p-*value < 0.1.

### 2.5 Statistical analysis and figure representation

Figures for methylome and transcriptomic results were generated using the *ggplot2* and *DOSE* R packages. Schematic figures were created using the BioRender app.

## 3 Results

### 3.1 HIV-Tat expression results in altered DNA methylation and RNA transcription

**Figure 1:**
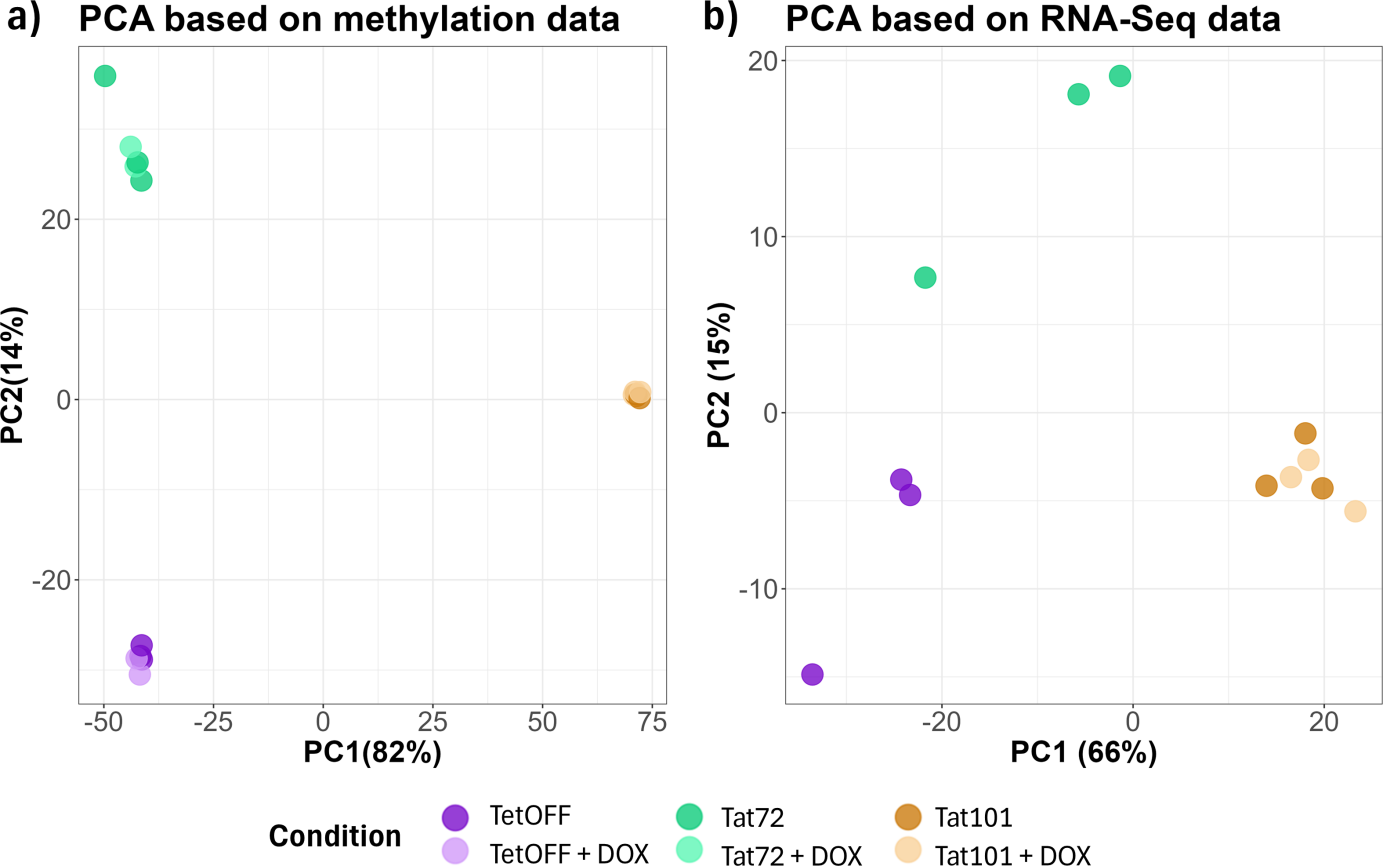
Principal component analysis (PCA) of DNA methylation data and RNA-Seq data between different Tat-expressing Jurkat cell lines and doxycycline treatment. **(A)** β-Values of all CpG in each sample were considered to calculate the PC. **(B)** Transcripts per million reads in each sample were considered to calculate the PC. DOX condition was only performed in the Tat101 Jurkat cell line. The percentage of variance of each PC is shown between brackets. DOX, doxycycline; PC, principal component.

To determine the effect of Tat protein on DNA methylation, we compared genome-wide methylation patterns between Jurkat cell lines stably expressing full-length Tat protein (101aa; Tat101), Tat’s first exon (72aa, Tat72), and cells stably transfected with an empty vector (TetOFF). Principal component analysis (PCA) revealed a high homogeneity among replicates and a clear clustering based on the expression of different HIV Tat protein constructs (**Figure 1A**). PC1 explains 82% of the variance of methylation data showing that Tat101 presents a more differentiated methylation profile than that obtained from Tat72 and TetOFF cell lines, which show less variability between them. To evaluate whether changes in methylation induced by Tat were restored after its removal, we switched off Tat expression by adding 1 μg/mL doxycycline (DOX). We observed no differences between the different cell lines in the presence or absence of DOX, suggesting that, once established, the methylation pattern is not reversed upon Tat silencing (**Figure 1A**). Subsequently, we assessed the transcriptomic profile by RNA-Seq to describe the effects of Tat on the transcription status of the cell. We also inspected transcriptional data with a PCA revealing clusters based on Tat protein expression (**Figure 1B**), with PC1 explaining 66% of the variance, with full-length Tat representing a clearly different RNA expression profile. Transcriptional patterns in Tat101 do not show a high variability upon DOX treatment. PCA clusters based on transcriptional data group samples similarly to those obtained with methylation data. Collectively, these data suggest that Tat101 expression results in both methylation and transcriptomic changes that persist despite DOX treatment.

### 3.2 DNA methylation changes were induced by full-length HIV-Tat protein

Full-length Tat expression resulted in an increased median β-value of 0.841 (0.840–0.841, 95% confidence interval) in Tat101 cell lines compared to 0.801 (0.799–0.802) and 0.785 (0.784–0.786) median β-values obtained in TetOFF and Tat72 cell lines, respectively (**Supplementary Figure 2**). This indicated that full-length Tat increased the DNA methylation status of the genome. According to the results obtained in the PCA, the median β-value of Tat101 before or after DOX treatment was nearly the same, 0.839 (0.839–0.840), indicating that Tat silencing did not change the DNA methylation status. To explore whether differences in DNA methylation were due to changes in the activity of DNA-methylating or demethylating enzymes, we assessed the activity of DNMT and TET enzymes and observed a slight (although not statistically significant) increase in the ratio of DNMT/TET activity in Tat101 compared to TetOFF (**Supplementary Figure 3**).

**Figure 2:**
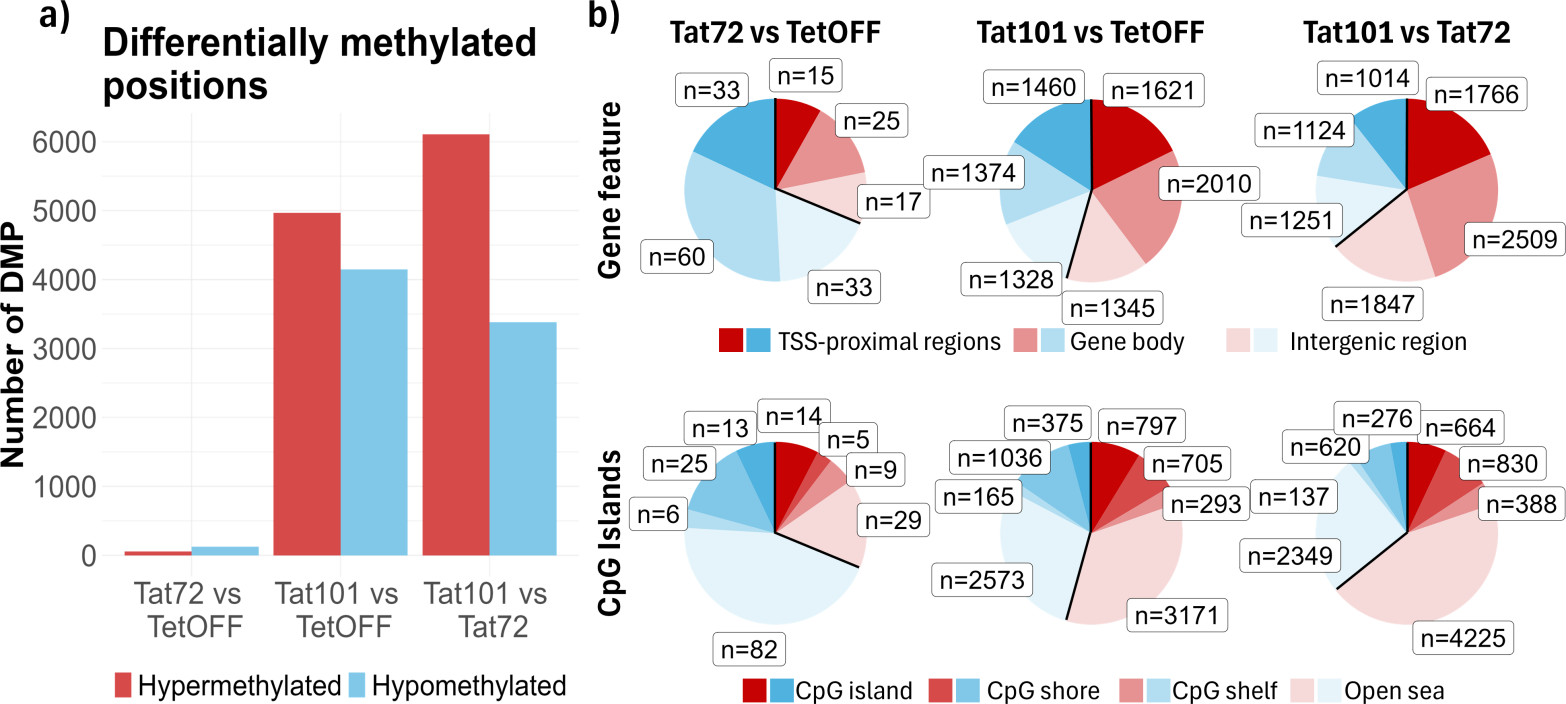
Characterisation of DNA methylation pattern at CpG level. **(A)** Bar plot with the number of significant DMPs (FDR < 0.05 and |Δβ| > 0.5) obtained from pairwise comparisons of different Jurkat cell lines transfected with Tat101, Tat72, or control plasmids. **(B)** Pie chart representing DMPs between conditions. DMPs were annotated based on two different genomic configurations: gene features (top), which include TSS proximal zone (1 to 5 kb upstream of the Transcription Start Site, Promoter, and UTR), gene body (exons, introns and 3′-UTR), and intergenic regions, and CpG island notation (bottom) based on CpG islands (CpG-dense 200-bp long region), CpG shores (1 bp to 2 kb away from a CpG island), CpG shelf (2 kb to 4 kb away from a CpG island), and open sea (>4 kb away from a CpG island). DMPs, differentially methylated CpG positions; FDR, false discovery rate. UTR, 5′-untranslated region.

To identify individual CpG positions that experience changes in DNA methylation, we performed a differential methylation analysis. We founded that the Tat72 cell line had 183 DMPs, of which 57 were hypermethylated and 126 were hypomethylated when compared to the TetOFF control cell line. In the Tat101 cell line, there were 9,116 DMPs, of which 4,967 were hypermethylated and 4,149 were hypomethylated in comparison to the TetOFF control cell line (**Figure 2A**). Due to the few changes observed between Tat72 and TetOFF, we also addressed whether Tat101 exerted the same methylation changes when compared to Tat72, finding 9,489 DMPs, 6,107 hypermethylated, and 3,382 hypomethylated DMPs, displaying a 63% of total DMPs in common to those observed between Tat101 and TetOFF. Additionally, in agreement with the previous results, there were no DMPs in the Tat101 expressing cell line after Tat silencing with DOX.

Furthermore, we examined the positions where DNA methylation changes occur, and we found that the DMPs were homogeneously distributed along all chromosomes, indicating that there were no preferred loci at the chromosome level where Tat101 induced these methylation changes (**Supplementary Figure 4**). We next focused on gene feature annotation (**Supplementary Figure 1**) and found that the proportion of DMPs located in TSS proximal regions, gene bodies, and intergenic regions was similar between cell lines in both differentially hypo- and hypermethylated CpGs (**Figure 2B**), suggesting that Tat elicited methylation changes near to gene structures. Meanwhile, the sum of DMPs annotated as part of CGIs, CGI shores, and CGI shelves showed similar proportions in methylation changes to that obtained in DMPs associated with TSS proximal regions since CGIs were normally associated with gene promoters (**Figure 2B**). The number of DMPs associated with CGI was much higher in Tat101 compared to TetOFF than in Tat72 compared to the same group. Altogether, this indicated that full-length Tat caused a higher number of methylation changes than its truncated form in DNA regions and that Tat may be regulating the expression of genes through this epigenetic mechanism.

### 3.3 Differentially methylated regions are preferentially located in the gene body and promoters

**Figure 3:**
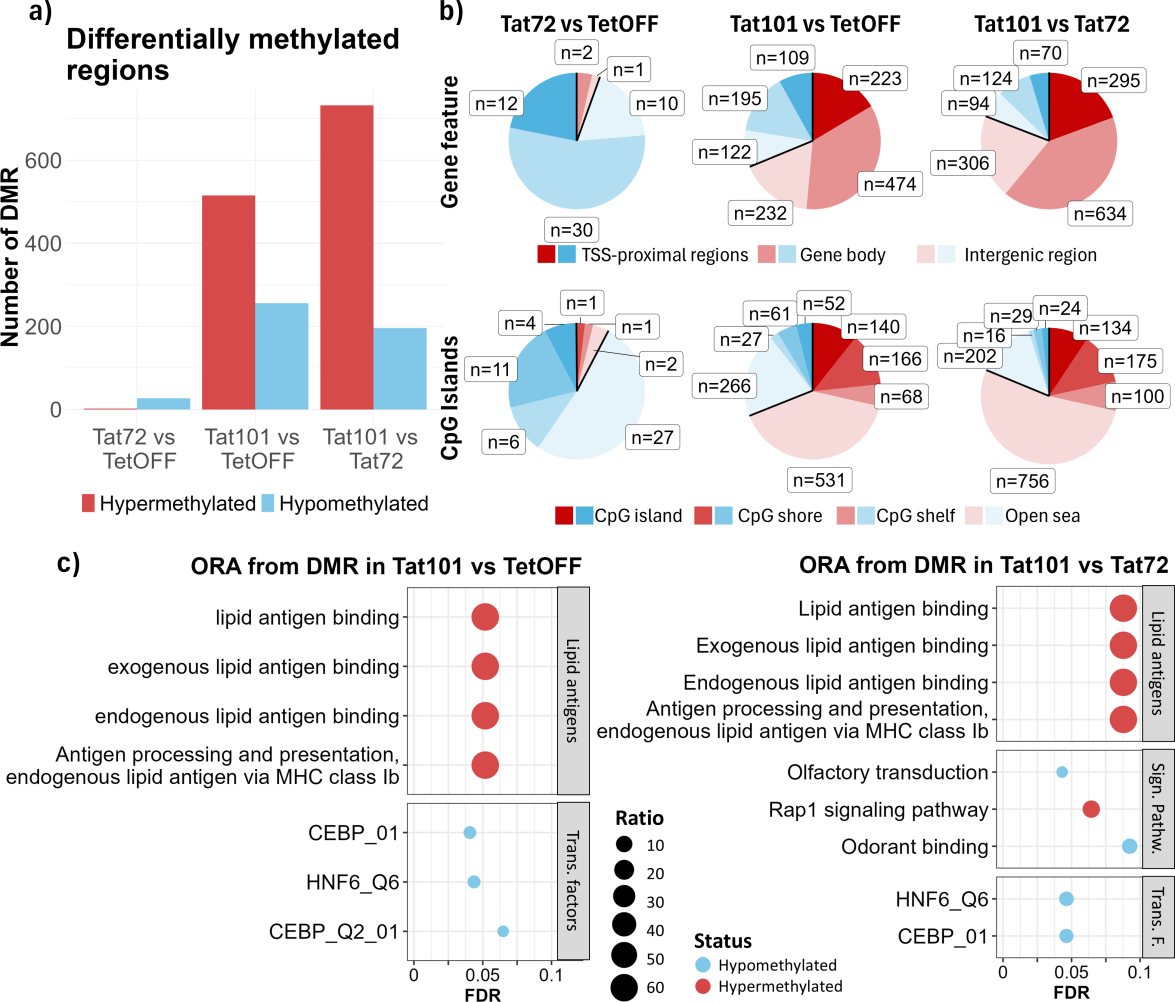
Characterisation of DNA methylation patterns at DMR level induced by distinct Tat protein constructs. **(A)** Bar plot with the number of DMRs (FDR < 0.1, |Δβ| > 0.25, and a minimum of 3 CpGs) resulting from pairwise comparisons. **(B)** Pie chart representing DMRs annotated to genomic features between conditions. DMRs were annotated based on two different genomic configurations: gene features and CpG island annotations (see **Figure 2** footnote). Some DMRs may annotate to more than one gene feature due to overlap. **(C)** Overrepresentation analysis of DMRs. The x-axis represents the FDR; the y-axis represents biological terms from Gene Ontology, KEGG, and TFT databases enriched with genes containing DMRs. The dot colour indicates hypomethylated (blue) or hypermethylated (red) DMRs in genes belonging to that term. Dot size (ratio) represents the proportion of altered genes in relation to the whole number of genes listed in that term. FDR, false discovery rate; KEGG, Kyoto Encyclopedia of Genes and Genomes; TFT, Transcription Factor Targets. DMR, differentially methylated region.

To evaluate the effect of methylation changes that could impact gene expression, we searched for DMRs, which we defined as genomic regions that contain at least three CpGs and a mean β-value difference of 0.25 between conditions. In agreement with the results observed in DMPs, there was a twofold increase in the number of hypermethylated over hypomethylated DMRs in the Tat101 vs. TetOFF comparison (724 and 335 DMRs, respectively) and about a 3.5-fold increase when comparing the Tat101 to Tat72 (970 and 275 DMRs). DMRs between Tat72 and TetOFF control cell lines were much lower than in other comparisons (3 hypermethylated and 40 hypomethylated in Tat72) (**Figure 3A**). According to the annotations established (**Supplementary Figure 1**), in general, we found two times more hyper-/hypomethylated DMRs in gene bodies (49%–50%) than in TSS proximal and intergenic regions (both a proportion of 24%–26%) in either Tat101 vs. TetOFF or Tat101 vs. Tat72 comparisons. In fact, 71% of hyper- and hypomethylated DMRs between Tat101 and TetOFF are also DMRs between Tat101 and Tat72. Hypermethylations in gene bodies (35%–42%) had a greater proportion compared with hypomethylations (8%–14%), like hypermethylations in intergenic regions (17%–20%) and TSS proximal regions (17%–19%) compared to hypomethylations in the same features (6%–9%) and (5%–8%), respectively, in both comparisons. Examining CGI annotation, we observed more hypo- and hypermethylated DMRs in open sea regions in all comparisons, followed by CGI shores. The proportion of DMRs in open sea regions was slightly higher in Tat101 when compared to Tat72 (65%) than when compared to TetOFF (59%) (**Figure 3B**).

We next identified genes containing DMRs (**Supplementary Table 1**) and performed an enrichment analysis to describe which biological processes may be affected by methylation changes (**Figure 3C**, **Supplementary Table 2**). The results showed the terms enriched in Tat101 when compared to TetOFF (left) or Tat72 (right). In both comparisons, the genes containing hypermethylated DMRs were mainly related to lipid antigen binding, processing, and presentation (*CD1B*, *CD1C*, and *CD1D*). In contrast, Tat101 cells were enriched in genes with hypomethylated DMRs affecting pathways under the regulation of key transcription factors such as CEBP and HFN6 (**Figure 3C**). These hypomethylated DMRs were located in genes related to immune and inflammatory responses such as *TNF*, *BNC2*, *CACNA2D3*, or *TSHZ3*.

### 3.4 Tat-induced DNA methylation alters the transcription of genes related to cell survival and immune response processes

**Figure 4:**
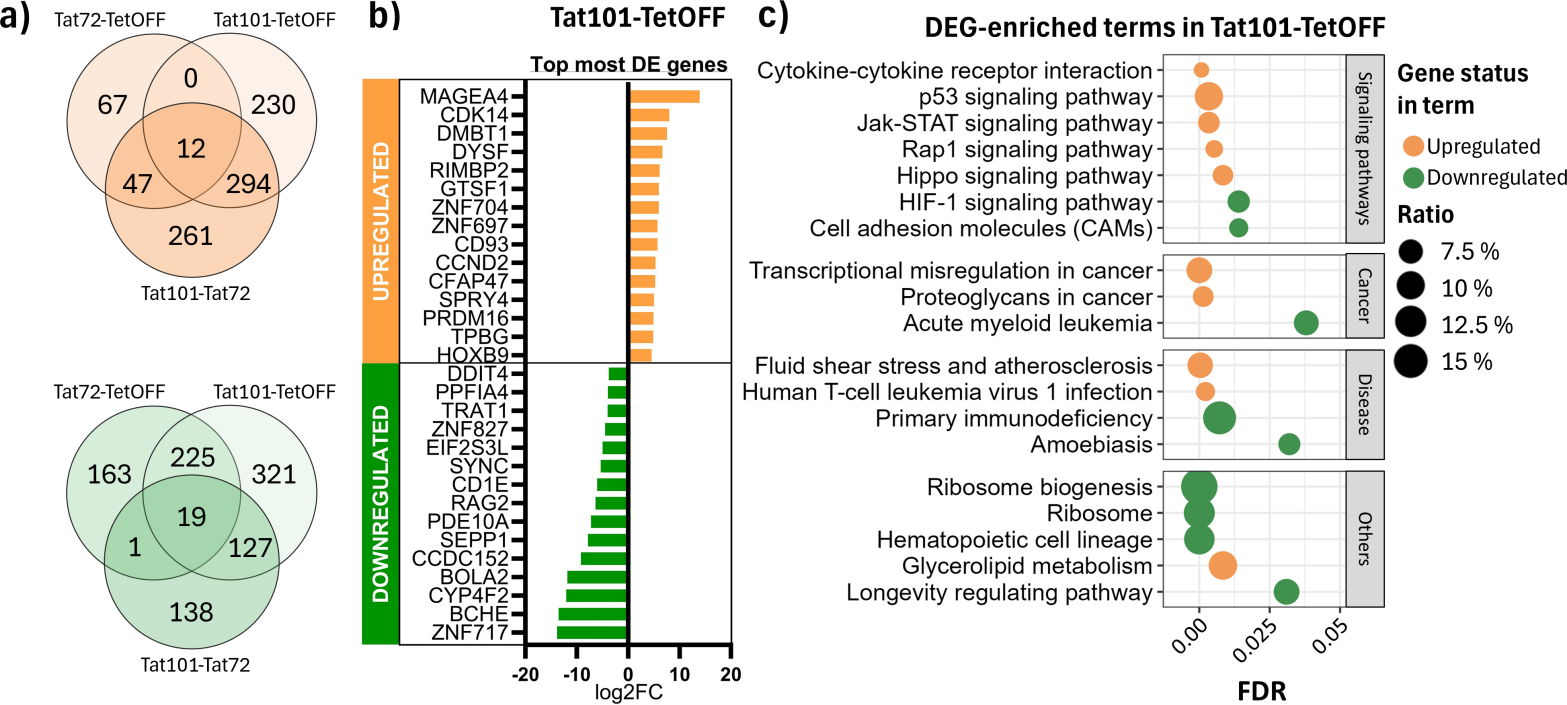
Differential expression analysis in RNA-Seq data. **(A)** DEGs in common among different comparisons. Venn diagrams depict overexpressed (orange) and repressed (green) DEGs in all contrasts. **(B)** Top 15 most differentially expressed protein-coding genes in Tat101 vs. TetOFF control cell line. **(C)** Overrepresentation analysis of all the DEGs in different Tat-expressing Jurkat cell lines. The x-axis represents the FDR; the y-axis represents biological terms from the KEGG database. Shape colour indicates downregulated (green) or upregulated (orange) DEG belonging to that term. Dot size (ratio) represents the proportion of altered genes in relation to the whole number of genes listed in that term. DEGs, differentially expressed genes; FDR, false discovery rate; KEGG, Kyoto Encyclopedia of Genes and Genomes.

To better understand how these changes in DNA methylation patterns impacted transcription, we performed RNA-Seq to evaluate the gene expression profile of each Tat-expressing cell line. We obtained a total of 1,275 differentially expressed (DE) genes (583 upregulated and 692 downregulated) in Tat101 compared to TetOFF, while when compared to Tat72, a total of 852 transcripts were DE (567 upregulated and 285 downregulated) (**Figure 4A**). In contrast to the results obtained in DNA methylation, the presence of Tat72 yielded 660 DE transcripts (126 upregulated and 534 downregulated) compared to TetOFF. These data showed that Tat72 can induce transcriptional changes when expressed; in particular, 256 altered genes were identical to those observed with Tat101 (12 genes upregulated and 244 downregulated genes), but the presence of full-length Tat induces an additional overexpression of 524 genes and the repression of 448 genes (**Figure 4A**). Of these genes, 43.3% are described also as DE between Tat101 and Tat72 (294 upregulated and 127 downregulated), suggesting that the presence of the second exon was key to the observed differential expression of such genes. The DEGs with a stronger difference between Tat101 and TetOFF were related to long non-coding RNAs (**Supplementary Table 3**). Here, we represent the top 15 protein-coding genes that were differentially expressed in this comparison (**Figure 4B**). Some of the most affected biological processes enriched in DE transcripts between Tat101 and TetOFF included signalling pathways that control cell cycle and survival as p53, JAK-STAT, or Hippo molecular cascades (**Figure 4C**, **Supplementary Table 4**).

**Figure 5:**
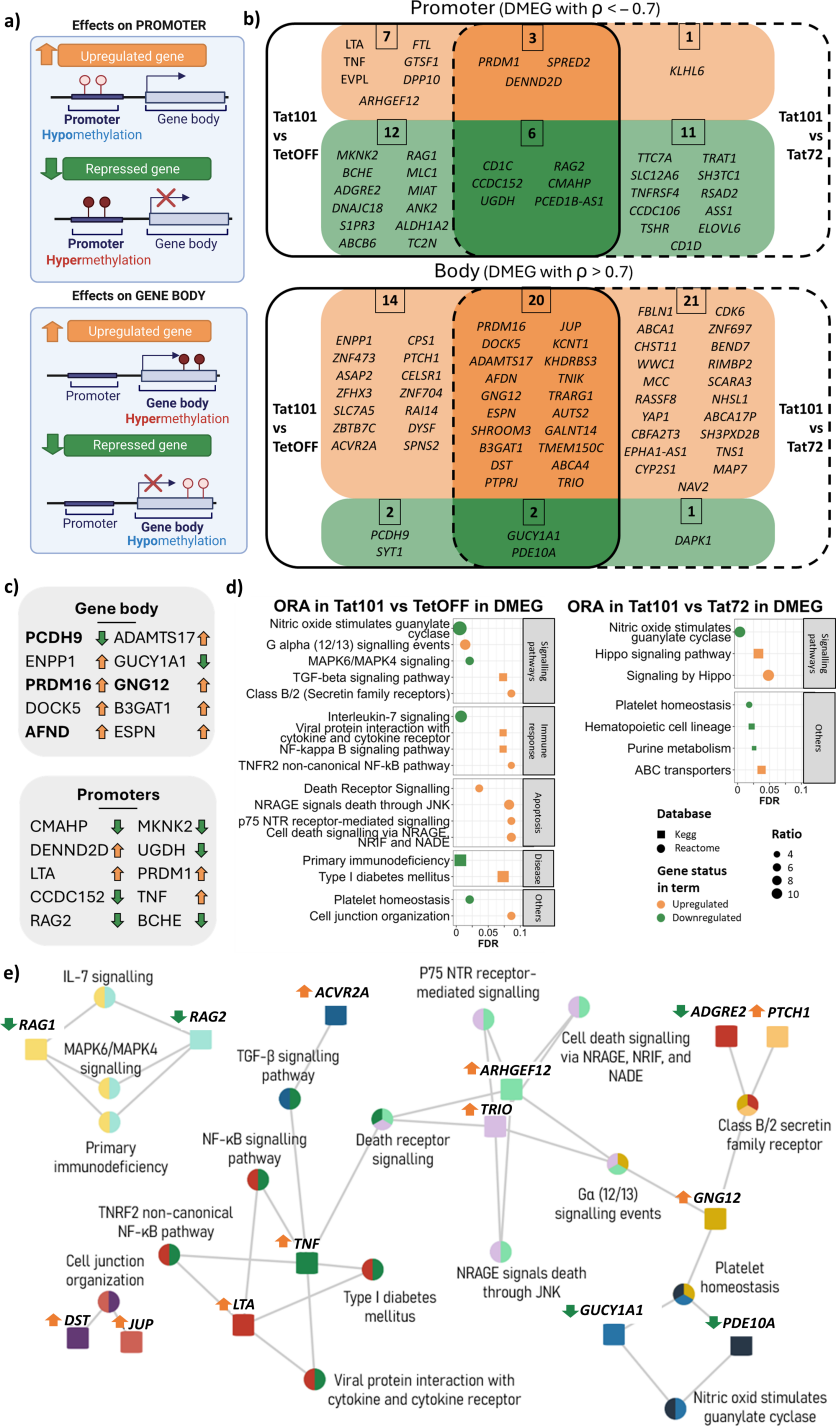
Differentially methylated and expressed genes (DMEGs) and biological processes affected by Tat101 expression. **(A)** Schematic representation of the relationship between promoter or gene body hyper-/hypomethylation and gene expression. **(B)** Venn diagram representing DMEGs Tat101 vs. TetOFF control cell line (left) or Tat72 (right). Genes depicted in orange are overexpressed and correlated to their methylation status in Tat101, while genes in green are downregulated under Tat101 condition. DMRs located in a TSS proximal region were inversely correlated (ρ < −0.7) with the expression values of that gene, and genes with a DMR placed in the gene body correlated positively (ρ > 0.7) with their expression level. **(C)** List of DMEG with the strongest correlation for promoters and gene body structures. The arrows at right indicate if genes are overexpressed (orange, upregulated) or repressed (green, downregulated) in Tat101 compared to TetOFF. Genes in bold contain more than one DMR (PCDH9 = x2, PRDM16 = x18, AFND = x2, and GNG12 = x2). **(D)** Overrepresentation analysis of all the DMEGs in different Tat-expressing Jurkat cell lines. The x-axis represents the FDR; the y-axis represents biological terms from KEGG and Reactome databases identified by squares and circles, respectively. Shape colour indicates downregulated (green) or upregulated (orange) DMEG belonging to that term. Dot size (ratio) represents the proportion of altered genes in relation to the whole number of genes listed in that term. **(E)** Biological terms and gene interaction network in overepresentation analysis (ORA) from Tat101 vs. TetOFF comparison. Squares, genes; circles, biological terms. Each colour represents a different gene. Arrows next to gene names are indicative of gene expression (orange, overexpressed; green, repressed) in Tat101 compared to TetOFF. DMRs, differentially methylated regions; TSS, transcription start site; FDR, false discovery rate; KEGG, Kyoto Encyclopedia of Genes and Genomes. ρ = spearmans coeficinent rho.

To understand the relationship between methylation and gene expression changes, we performed a correlation analysis using genes that contained differentially methylated regions and were differentially expressed (DMEG) in the presence of Tat protein. We considered a DMEG when the β-values of a DMR located in a TSS proximal region of a given gene were inversely correlated (ρ < −0.7) with the expression values of that gene. Genes with a DMR placed in the gene body with a positive correlation (ρ > 0.7) with their expression level (**Figure 5A**) were also considered DMEGs. This trend was confirmed when expression values were represented against methylation values (**Supplementary Figure 5**). In accordance with the general hypermethylation observed in Tat101 cells, a greater number of genes showed hypermethylated promoter regions and gene bodies and were associated with repression and overexpression of transcription of those genes, respectively (**Figures 5B, C**). Focusing on Tat101 methylation changes in the promoter regions, 10 DMEGs were hypomethylated and overexpressed compared to TetOFF control, which included members of tumour necrosis factor superfamily *LTA* and *TNF*, or *PRDM1* also known as *BLIMP1*. Among the 18 DMEGs hypermethylated in promoter regions and, therefore, downregulated genes were the immunoglobulin V-D-J recombining enzymes *RAG1* and *RAG2*, and the surface glycoprotein *CD1C*, implicated in the presentation of lipid antigens via MHC-Ib. Regarding the DMEG, four genes presented hypomethylation in their gene body and downregulation in gene expression, while 34 DMEGs were hypermethylated in their gene body and overexpressed, including *PRDM16*, *GNG12*, *TRIO*, or *AFND*. *PRDM16* had one of the strongest positive correlations between DMR β-values and its expression level, containing up to 18 hypermethylated DMRs in its gene body (**Figure 5C**). Furthermore, there were no DMEGs that contained DMRs in promoter and body regions simultaneously, suggesting that Tat only regulated gene expression through methylation in restricted regions of each gene.

Subsequently, we performed an enrichment analysis using all DMEGs to identify the biological pathways whose gene expression is altered by methylation changes induced by Tat (**Figure 5D**, **Supplementary Figure 6**). The terms enriched in such DMEGs in Tat101 when compared to TetOFF (left) or Tat72 (right) showed that full-length Tat protein was regulating nitric oxide-sensitive guanylyl cyclase and platelet homeostasis. Genes involved in immune response mechanisms and inflammation as NF-κB signalling (*LTA* and *TNF*), TGF-β signalling pathway (*TNF* and *ACVR2A*), and apoptosis-related terms (*ARHGEF12*, *TNF*, and *TRIO*) were overexpressed in Tat101 cell line when compared to TetOFF, while *RAG1* and *RAG2* genes, associated with IL-7 signalling and MAPK6/MAPK4 signalling, were repressed (**Figure 5E**). Moreover, in the comparison of Tat101 with Tat72, the full-length protein upregulated the expression of genes that belong to the Hippo (*WWC1* and *YAP1*) pathway associated with cell proliferation and apoptosis (**Figure 5E**). Taken together, these data showed that Tat101 regulated the expression of genes involved in inflammation, immune response, and survival through the modification of the methylation status of the promoter and the gene body.

## 4 Discussion

HIV infection and specifically Tat protein are known to alter epigenetic mechanisms such as chromatin acetylation/methylation or non-coding RNA expression (11, 33, 42), but whether Tat alters the DNA methylation landscape of CD4 T cells has not yet been explored. In this study, we described the role of Tat in altering the methylation landscape of host cells and its relationship with gene transcriptional regulation upon Tat expression. Previously, other authors reported that HIV-1 infection reshapes the host DNA methylation landscape in PWH, leading to alterations in gene expression that favour not only viral replication but also accelerated ageing (21, 43, 44). Moreover, another study described that murine neuroglia models exposed to HIV-Tat suffer changes in their DNA methylation patterns, consistent with the findings of this study, contributing to neurological manifestations of HIV-1 pathology (45, 46). Our experimental approximation focuses on how intracellular HIV-Tat protein alters the DNA methylation landscape in a Jurkat T-cell model and how these methylation changes affect gene transcription in such cells. We used an extensively characterised Jurkat TetOFF cell model, shown to mimic Tat levels induced by NL4.3 infection of MT-2 cells (42), allowing us to accurately measure changes induced by either full-length Tat or its first exon-only counterpart without the interference of other viral products. We have shown that the expression of full-length Tat resulted in DNA methylation changes inducing 9,116 DMPs, regardless of whether HIV-Tat was silenced upon 48 hours of DOX treatment, suggesting that changes in DNA methylation were stable and not easily reversible under our experimental conditions. DNA methylation changes are usually maintained in the absence of the inductor, which is known as epigenetic memory (47, 48). On top of that, Esteban-Cantos et al. reported that the methylation changes induced by HIV infection only revert partially after 1 year of ART administration (21), supporting our results that methylation changes induced by Tat are stable upon DOX treatment. The magnitude of the number of changes induced in the presence of full-length Tat compared to the Tat72 construct indicated that the second exon was required to alter the DNA methylation landscape, which may eventually impact gene expression. DNMT and TET enzymes could be found within cellular factors recruited to the transcription complex that may be influenced by Tat101. The lack of the second exon in Tat72 may limit recruitment or difficulty in the activity of DNMT enzymes, which would then result in reduced hypermethylated DMPs/DMRs in Tat72. This would reflect a greater number of hypermethylated DMPs/DMRs in the Tat101 vs. Tat72 comparison than in the Tat101 vs. TetOFF comparison. Specifically, the importance of Tat’s second exon in complementary processes in HIV-1 infection has been previously documented. Additional non-transcriptional functions exhibit significant dependence on Tat’s full-length protein compared to the truncated form of Tat72, including NF-κB, NF-AT-, and Sp1-dependent transcriptional activities, cytoskeleton-related functions, and CD4 and CD1 cell surface expression (4, 8). However, the necessity of the second exon’s presence to alter certain processes through DNA methylation in human immune cells has not been described so far.

We observed an overall hypermethylation status in Tat101, both at the CpG level and (more prominent) at the DMR level. Several studies reported an increase in DNMT activity after Tat expression. Increased DNMT activity can result in altered DNA methylation levels (49). However, we did not detect a clear increase in this direction after Tat expression, which may be due to the nature of the Jurkat T cell model (32–34, 50). The number of changes associated with potentially regulatory regions (CGI or TSS proximal zones) is much higher in Tat101 compared to either the control or Tat72, meaning that full-length Tat protein likely played a crucial role in regulating gene expression through the methylation of key regions controlling transcription. Particularly, DMRs located in TSS proximal regions or gene bodies were proportionally higher than the CpGs annotated with the same features. This suggests that Tat101 elicits methylation changes directed to DNA sequences controlling gene expression. While the underlying mechanism to induce these methylation changes is not well characterized, three different mechanisms of Tat–gene interactions have been defined: 1) transcriptional activation through the interaction with TAR-like sequences in newly formed mRNAs (51, 52), 2) transcriptional modulation of the target gene by binding to the promoter region (53–56), and 3) regulation of transcription via interaction with transcription factors (11, 57, 58). This HIV-induced epigenetic change modifies cellular functions contributing to pathogenesis. We found Tat-induced methylation variations in genes such as *TNF*, *CD1C*, and *CD1D*, belonging to pathways involved in the binding, processing, and presentation of lipid antigens, immune response, and inflammatory pathways, which are processes well-documented during HIV-1 infection (4, 59).

In addition to DNA methylation, several other mechanisms can regulate gene expression, such as histone modifications (acetylation, methylation, and phosphorylation), chromatin remodelling, or non-coding RNAs (microRNAs and long non-coding RNAs) (14, 60, 61). Together, these diverse mechanisms allow for intricate control over gene expression in response to various cellular signals and environmental changes. The complex interaction between HIV-Tat and host epigenetic mechanisms is not well studied. Thus, immunoprecipitation or co-immunoprecipitation experiments may answer whether DNMT enzymes may be recruited to Tat transcriptional complexes. Additional experiments using ATAC-seq could help identify differences in chromatin accessibility in Tat-expressing cell lines. Therefore, it should be assessed whether Tat acts by the recruitment of RNA polymerase II to target genes and interact with transcription factors (11) or/and interact with the chromatin remodelling machinery (62) or DNMT complex to alter gene expression. Previous research employed organ-specific transgenic mice models to study modifications in DNA methylation, such as the brain (33, 63) or heart (64). These studies, while valuable, are limited to specific tissues, whereas our findings provide a broader perspective on Tat’s epigenetic effects across genomic DNA. HIV-1-encoded proteins interact with chromatin remodelling complexes and histone-modifying enzymes, and specifically, HIV-Tat inhibitors can reverse Tat-induced chromatin alterations, further reinforcing the role of Tat as a driver of epigenetic changes (65, 66). In our study, Tat influenced gene expression through DNA methylation in 5.1% of the DEG of Tat101 cells, compared to TetOFF cells. Understanding the relationship between Tat-directed DNA methylation and gene expression is essential for elucidating the mechanisms of gene regulation in HIV infection. As reported by others, there is a negative correlation with gene expression when methylation changes occur in the promoter region (67) and, by contrast, a positive correlation when methylation changes occur in the gene body (17). Our correlation data (**Supplementary Figure 5**) indicate that this is the case in our cellular model. We show that the general hypermethylation pattern elicited by Tat101 leads to a higher number of downregulated genes when methylation occurs in promoters. Conversely, Tat leads to a greater number of overexpressed genes when hypermethylation is found in gene bodies. Moreover, we did not find concurrent methylation modifications in both promoter and gene body from the same gene, which suggests that the alteration of only one feature is enough to alter gene expression and that the modifications in one specific region are a highly coordinated and regulated process (19).

Intersecting methylation and expression data, we found that Tat101 influenced the expression of 64 genes through methylation modifications compared to TetOFF. The expression of *CD1C* and *CD1D* genes related to lipid antigen binding and presentation were downregulated, while their promoters were hypermethylated. The repression of these genes has been previously described in TetOFF (4) and TetON (11) models of stable Tat expression and in Jurkat cells upon HIV-1 infection (68). Kelly et al. proposed that HIV-1 fosters the downregulation of *CD1C* to evade the immune system, hindering CD1c-restricted T-cell response and their production of IFN-γ (68). This would represent an important pathogenic mechanism by which Tat would contribute to sustaining HIV infection by inhibiting specific immune responses. PRDM16 methylation was highly altered in the presence of Tat, more than in other genes, with 18 DMRs. This gene plays a crucial role in lipid metabolism, particularly in the regulation of adipose tissue function and the formation of beige adipocytes (69). Other genes were downregulated due to hypermethylation in their promoter regions including *RAG1* and *RAG2*. In a Tet-ON Jurkat cell model of Tat expression, the *RAG1* gene was also downregulated (11). The absence of these genes impairs the immune system’s ability to effectively recognise and respond to viral pathogens, leading to increased susceptibility to severe viral infections (70). The NF-κB signalling pathway is critical in mediating inflammatory responses, and several reports show how Tat alters NF-κB signalling and viral gene expression (8, 71, 72), and a sustained activation is associated with chronic inflammation, immune activation, tissue damage, and an increase in comorbidities (73). We found an upregulation in *LTA* and *TNF* gene expression, which goes along with an increased hypomethylation on its gene promoters. Several genes included in the altered pathways in the presence of Tat have been linked to cellular ageing and senescence, which is closely related to the presence of comorbidities. In addition, we also show an upregulation of *TNF* and *ACVR2A* genes, which belong to the transforming growth factor-beta (TGF-β) pathway. TGF-β is secreted by senescent cells and mediates paracrine senescence as a component of the senescence-associated secretory phenotype (SASP). TGF-β signalling contributes to tissue remodelling and chronic inflammation, commonly seen in age-related diseases (74). We also observed an upregulation in triple functional domain protein (*TRIO*) and ADAM Metallopeptidase with Thrombospondin Type 1 Motif 17 (*ADAMTS17*) genes together with a gene body hypermethylation. These alterations favour anti-apoptotic processes (75, 76), and thus, the enrichment analysis shows altered cell death signalling pathways. Future experiments, such as the knockout or knockdown of these genes or the blockade of their signalling routes, will help determine whether the presence of Tat still alters the expression of these key inflammatory markers and apoptotic indicators when these genes are knocked out.

Despite these important findings, it is unclear how Tat induces hypermethylation and alters CpG status. Given previous evidence on different cellular models, a plausible explanation is that Tat interacts directly with DNA methylation enzymes or alters complex pathways involving other cellular factors, resulting in DNA-methylation changes. Using the Jurkat cell line to study the alteration of biological processes induced by Tat has limitations due to its transformed origin and genetic alterations, which may not accurately reflect the HIV infection process in non-transformed cells. However, this model allows us to study the isolated effect of Tat protein in the alteration of methylation patterns. The full-length Tat protein induces significant epigenetic changes, such as global hypermethylation and altered gene expression, indicating potential therapeutic targets associated with comorbidities and HIV-related accelerated ageing in PWH. Abnormal methylation patterns have been associated with cell senescence and age-related comorbidities (77, 78). Age-related comorbidities are more prevalent in PWH than in the general population, possibly as a result of cellular senescence and the inflammation it causes (79–81). Moreover, a recent publication has demonstrated that the use of a Tat inhibitor didehydro-cortistatin A improves the inflammatory environment induced by the virus, altering the methylation landscape of *ex vivo* treated cells (82). Understanding Tat’s influence on DNA methylation and gene expression can lead to interventions aimed at reverting aberrant epigenetic changes, reducing inflammation and cellular senescence, and improving the health outcomes for PWH. This research deepens our knowledge of how HIV-1 interacts with host cellular machinery at an epigenetic level, providing insights into the broader impacts of viral infections on ageing and disease processes. This could pave the way for further studies on epigenetic regulation in other chronic infections and inflammatory conditions. This study reveals that the HIV-1 Tat full-length protein led to global hypermethylation in Tat-expressing cells. This change in methylation status partially influenced gene expression, impacting genes involved in inflammation, apoptosis, and biological processes implicated in the viral replication cycle, which are also associated with cellular senescence and accelerated ageing in immune cells.

## Data Availability

The datasets presented in this study can be found in online repositories. The names of the repository/repositories and accession number(s) can be found below: https://www.ncbi.nlm.nih.gov/geo/query/acc.cgi?acc=GSE282546, GEO.
