# Supplementary material for: Intracellular HIV-1 Tat regulator induces epigenetic changes in the DNA methylation landscape
Source: Front Immunol. 2025 Mar 4;16:1532692. doi: 10.3389/fimmu.2025.1532692 (PMC11913862; doi:10.3389/fimmu.2025.1532692)

Supplementary Material

# Supplementary Data

**Supplementary Figure 1:** Two different annotations used to classify the position of DNA methylation changes in the genome.

**Supplementary Figure 2:** Beta values distribution in Jurkat cell lines transfected with different Tat constructions (Violin plot).

**Supplementary Figure 3:** DNMT/TET enzymatic activities in Tat transfectant Jurkat cell lines.

**Supplementary Figure** **4**: Epigenomic methylation differences obtained in each comparison at chromosome positions (Manhattan plots)

**Supplementary Figure 5**: Scatter plot showing the transcription level in TPM versus methylation level in β values.

**Supplementary Figure 6**: Over representation analysis of the DMEGs in different Tat-expressing Jurkat cell lines using Gene Ontology as reference data base.

**Supplementary Table 1.** Differentially methylated genes in Tat101 vs TetOFF.

**Supplementary Table 2.** Over Representation Analysis with genes containing DMR.

**Supplementary Table 3.** Differentially expressed genes in Tat101 vs TetOFF.

**Supplementary Table 4.** Overrepresentation analysis result table with differentially encoding genes from Tat101-TetOFF comparison.

## Supplementary Figures


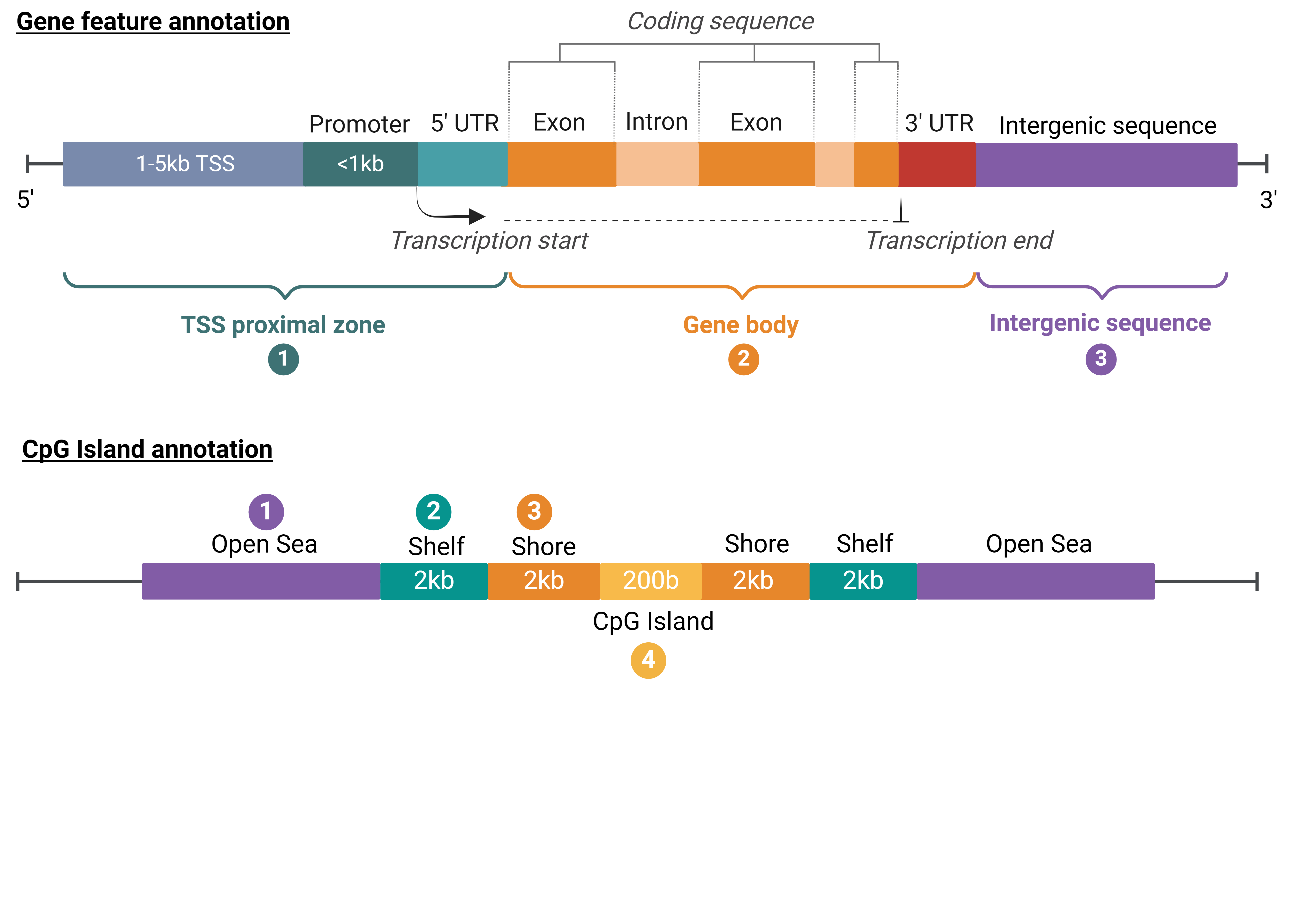
**Supplementary Figure 1**. Two different annotations used to classify the position of DNA methylation changes in the genome.

*
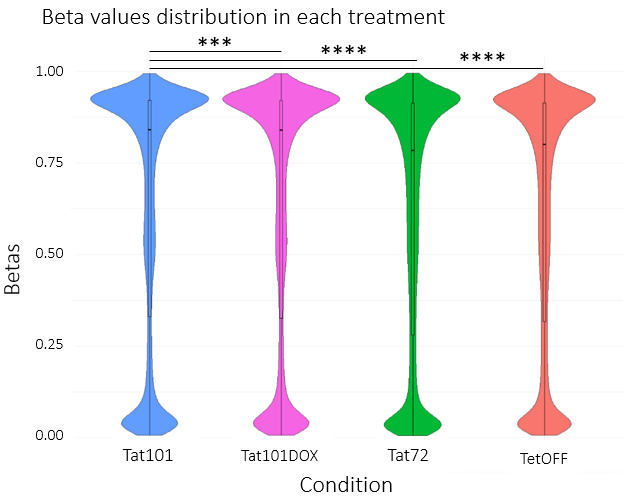
*

**Supplementary Figure 2**. β-values distribution in Jurkat cell lines transfected with different Tat constructions. Box plots within the violin shapes indicate 25, 50 and 75 percentiles. Wider areas in the violin represent a higher presence of CpG reaching the given β-value. Differences between β-values were assessed by Wilcoxon test, obtaining a *p*-value < 10^-16^ (****), except for Tat101-Tat101DOX (*p*-value < 10^-5^ (***).


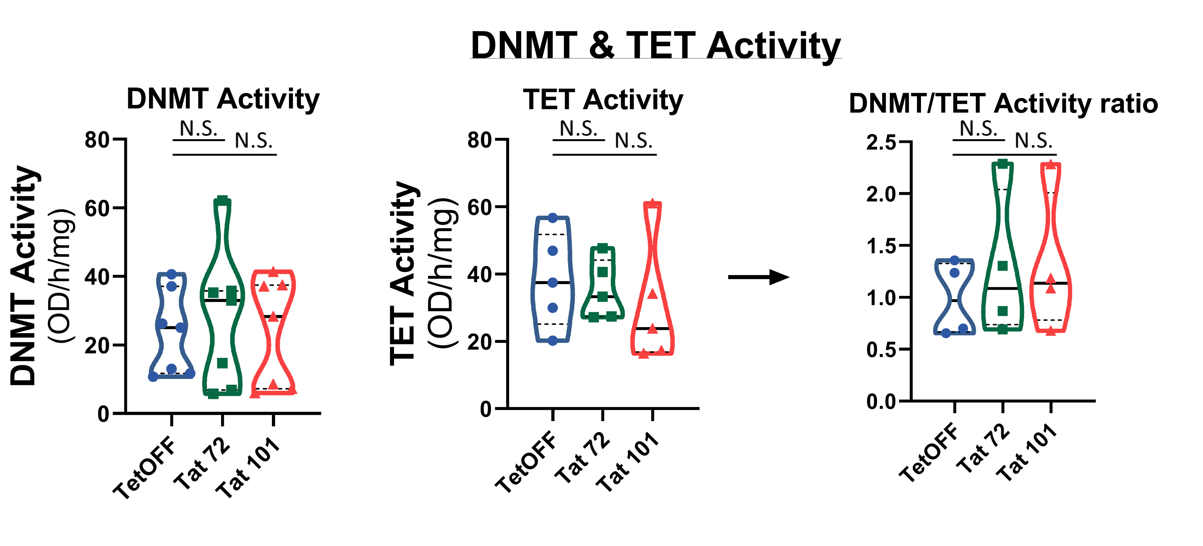
**Supplementary Figure 3.** DNMT and TET activity in Tat-expressing cells. DNMT and TET enzyme Activity in Jurkat cell lines with different forms of HIV Tat protein and its ratio. Nuclear extracts were isolated from Jurkat cell lines to determine the overall DNMT and TET activity. We used EpiQuik™ Nuclear Extraction kit (Epigentek, NY, USA, Ref. OP-0002) to obtain nuclear extracts. To determine DNMT and TET activity we used Colorimetric EpiQuik™ DNMT Activity/Inhibition Assay Ultra kit (Epigentek, NY, USA, Ref. P-3001), and Colorimetric Epigenase™ 5mC Hydroxylase TET Activity/Inhibition Assay Kit (Epigentek, NY, USA, Ref. P-3086) adding 2.2 to 5.32 µg of total protein respectively and using a wavelength of 655 nm. Final activity was calculated applying the formulas given in the manufacturer's instructions. Data are expressed as OD/h/mg. Ratio was calculated between paired samples. For Statistical analysis of Enzyme activity, GraphPad Prism (version 10.2.0) was used. Data are presented as median with interquartile range of five and seven independent experiments in duplicate in Activity assays. Comparisons between groups were made using Ordinary one-way ANOVA (repeated measures). No significant differences were observed setting the *p-*value at 0.05.


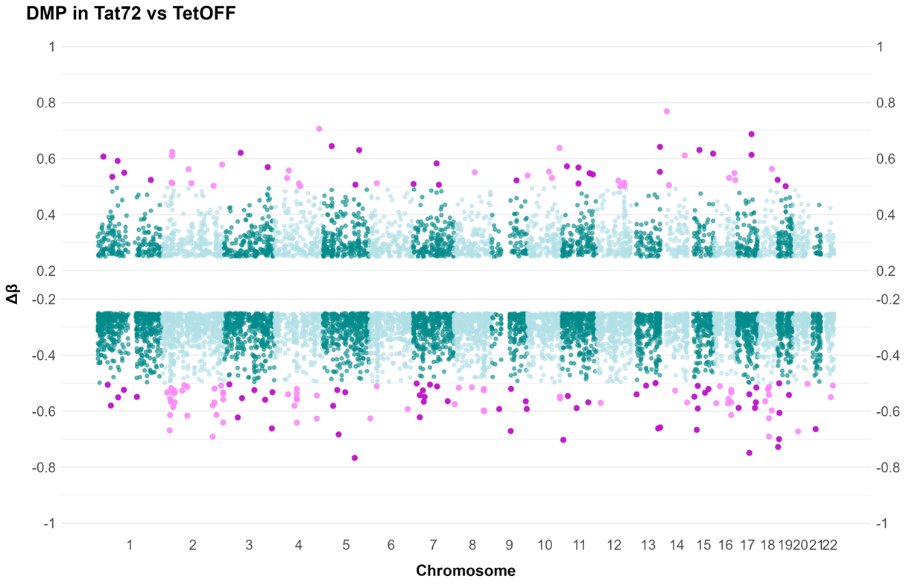

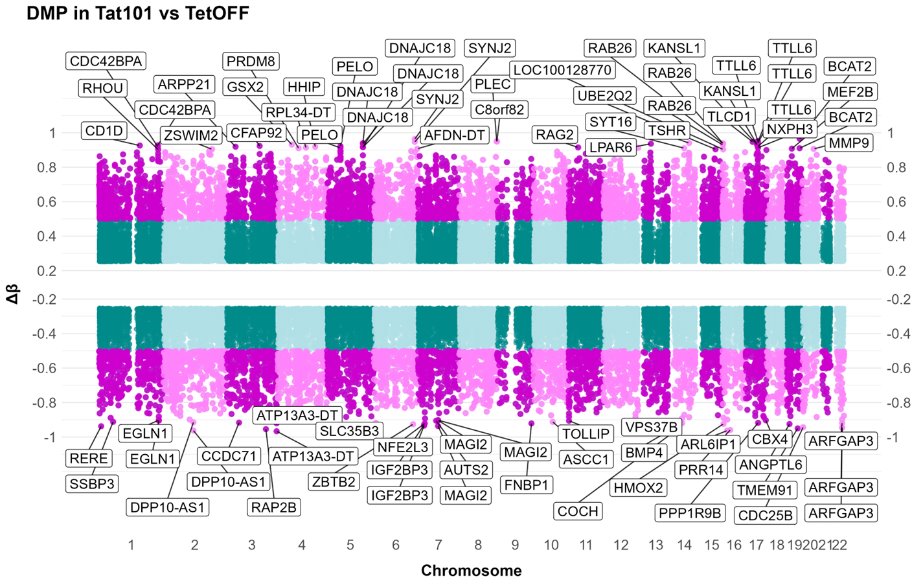


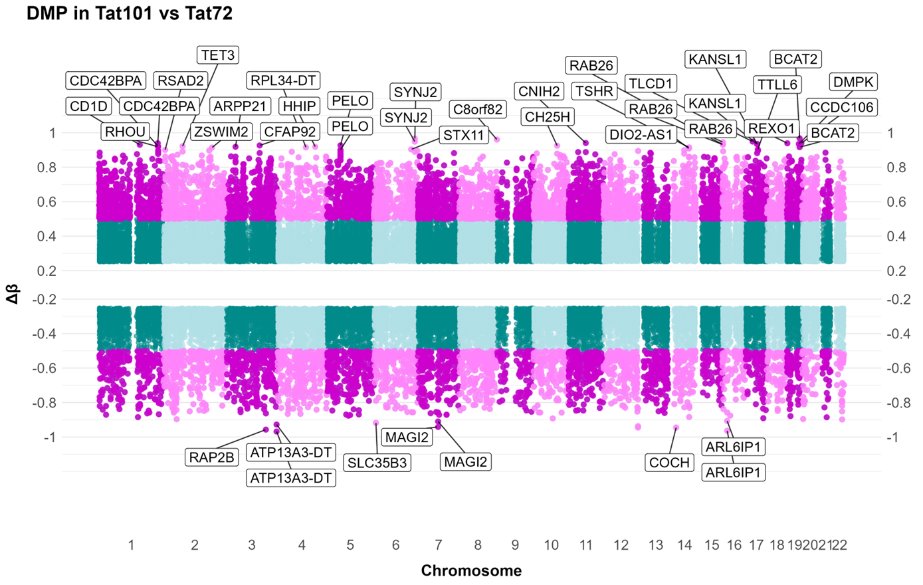


**Supplementary Figure 4.** **Epigenomic methylation differences obtained in each comparison at chromosome positions** Each dot represents an individual DMP. The x axis represents the chromosomic location of DMPs along each chromosome and y axis represent the increment of β (Δβ) of the first group over the reference. Dots with |Δβ| > 0,5 are shown in pink/purple. Gene labels correspond to DMP with |Δβ| > 0,9. **(A)** Tat72 vs. TetOFF comparison. **(B)** TetOFF vs. Tat101 comparison. **(C)** Tat101 vs. Tat72 comparison. Δβ are calculated taking the β value of each CpG in the second group from the first one in each comparison.


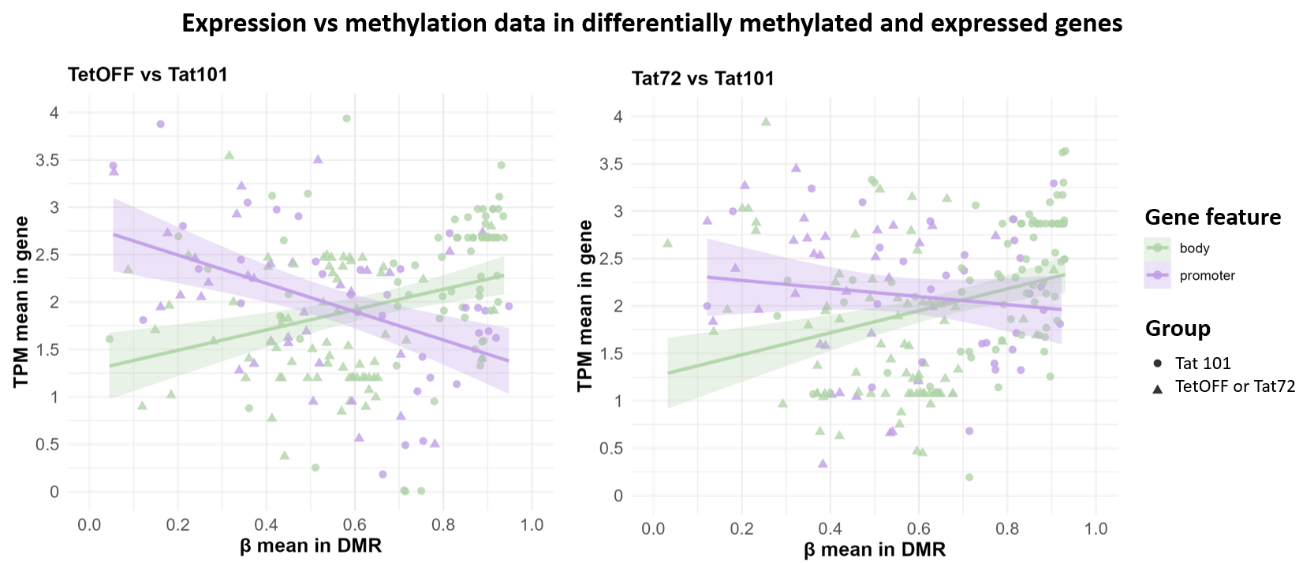
**Supplementary Figure 5.** **Scatter plot showing the transcription level in TPM (y-axis) versus methylation level in β-values (x-axis).** Dots represent all the biological replicates for each DMEG. Regression lines are calculated for direct correlation of expression and methylation level at gene body (green) and for inverse correlation between expression and methylation level at gene promoters (purple).

**Supplementary Figure 6.** **Over representation analysis of the DMEG** in different Tat-expressing Jurkat cell lines. The x-axis represents the FDR; y-axis represents biological terms from Gene Ontology: Biological Process database. Dot colour indicates downregulated (green) or upregulated (orange) DMEG belonging to that term. Dot size (ratio) represents the proportion of altered genes in relation to the whole number of genes listed in that term.


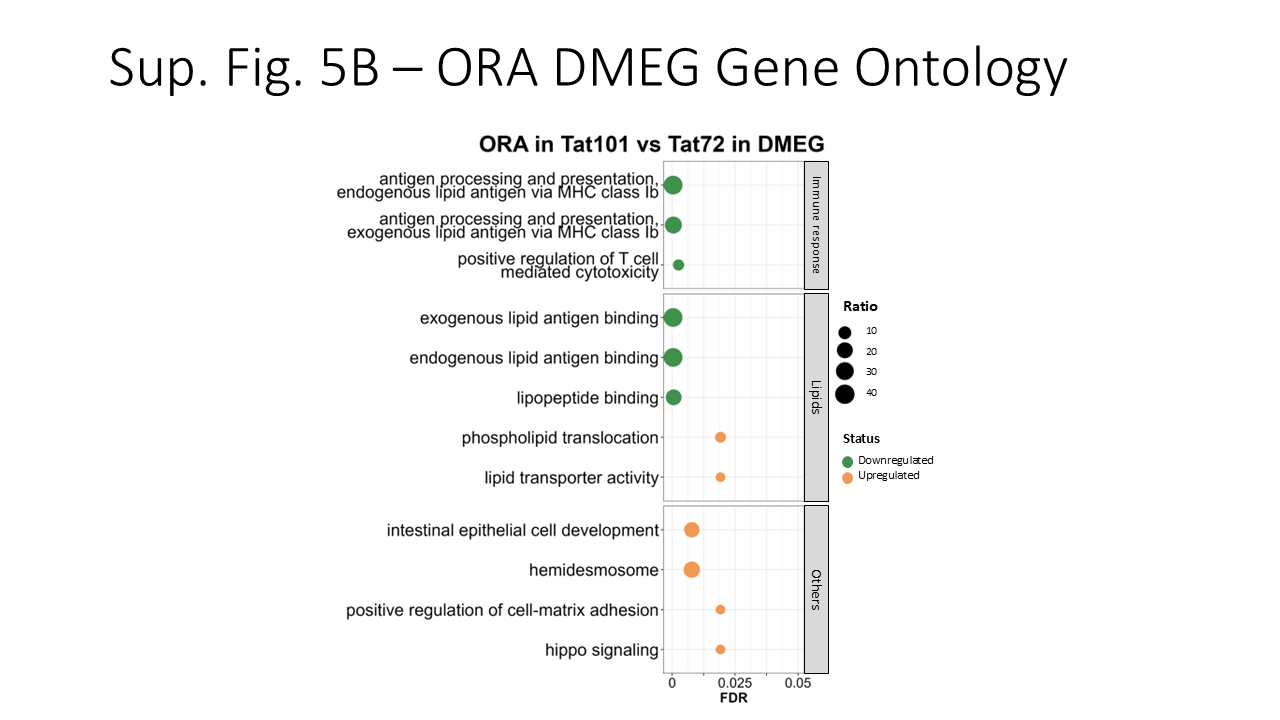

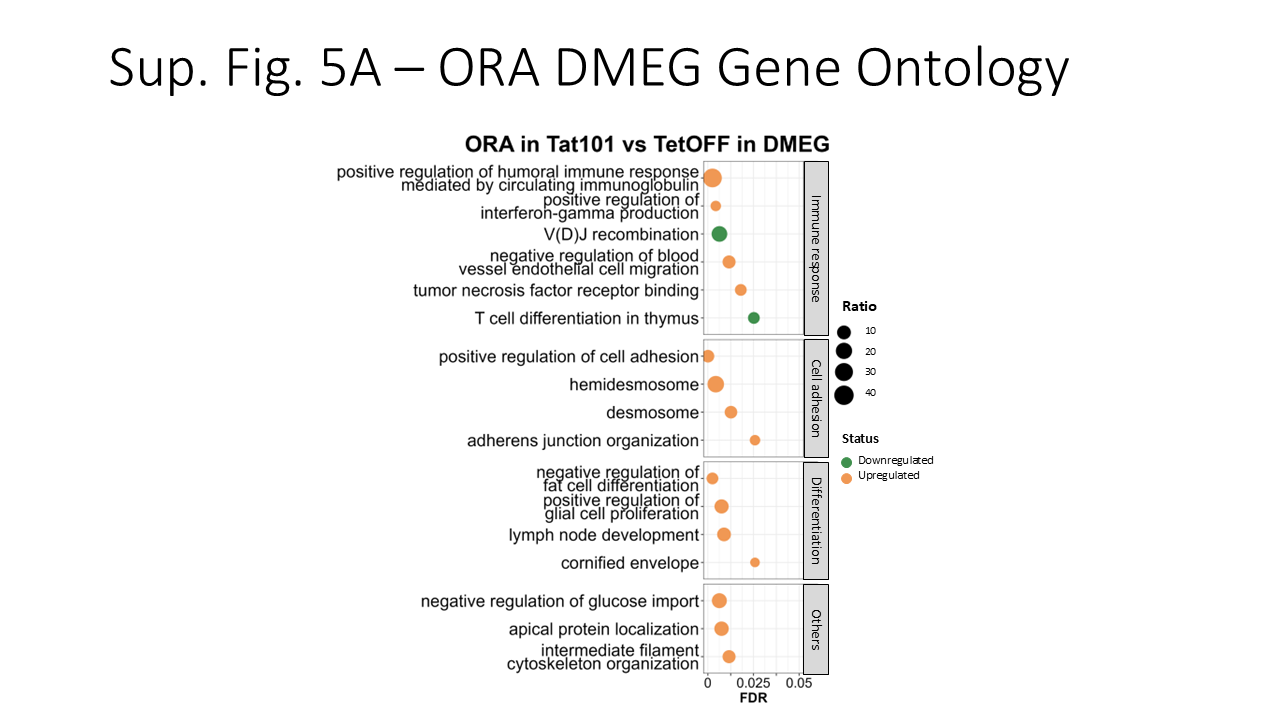

Supplement: Supplementary file 1 [file DataSheet1.zip › Supplementary_Material.docx]
